# Complete genome sequence of *Methanosphaera* sp. ISO3-F5, a rumen methylotrophic methanogen

**DOI:** 10.1128/mra.00043-24

**Published:** 2024-03-01

**Authors:** Nikola Palevich, Jeyamalar Jeyanathan, Kerri Reilly, Faith P. Palevich, Paul H. Maclean, Dong Li, Eric Altermann, William J. Kelly, Sinead C. Leahy, Graeme T. Attwood, Ron S. Ronimus, Gemma Henderson, Peter H. Janssen

**Affiliations:** 1 AgResearch Ltd., Grasslands Research Centre, Palmerston North, New Zealand; 2 Laboratory for Animal Nutrition and Animal Product Quality, Department of Animal Sciences and Aquatic Ecology, Faculty of Bioscience Engineering, Ghent University, Ghent, Belgium; 3 AgResearch Ltd., Hopkirk Research Institute, Palmerston North, New Zealand; 4 School of Veterinary Science and Centre for Bioparticle Applications, Massey University, Palmerston North, New Zealand; 5 Blue Barn Life Sciences Ltd., Feilding, New Zealand; 6 New Zealand Agricultural Greenhouse Gas Research Centre (NZAGRC), Palmerston North, New Zealand; 7 Bacthera AG, Basel, Switzerland; Portland State University, Portland, Oregon, USA

**Keywords:** *Methanosphaera*, methanogen, methane, methanol, ruminant, genome

## Abstract

*Methanosphaera* spp. are methylotrophic methanogenic archaea and members of the order Methanobacteriales with few cultured representatives. *Methanosphaera* sp. ISO3-F5 was isolated from sheep rumen contents in New Zealand. Here, we report its complete genome, consisting of a large chromosome and a megaplasmid (GenBank accession numbers CP118753 and CP118754, respectively).

## ANNOUNCEMENT

Members of the genus *Methanosphaera* produce methane from methanol and hydrogen and occur in the rumen (1, 2) and other gut environments (3). *Methanosphaera* sp. ISO3-F5 was isolated from a rumen sample collected from a ruminally fistulated wether sheep grazed on a perennial ryegrass (*Lolium perenne*) and white clover (*Trifolium repens*) mixed pasture in New Zealand (−40°21′22.90″S, 175°36′ 40.07″E). Briefly, ISO3-F5 was isolated from rumen contents via dilution-to-extinction cultivation (4) in basal medium with yeast extract (BY^+^) at 39°C (5) as described by Jeyanathan (6). Subcultures were supplemented with antibiotics [streptomycin (10 µg/mL), ampicillin (10 µg/mL), and vancomycin (86.7 µg/mL)] then heat treated at 50°C for 30 min to eliminate surviving bacteria.

For genomic sequencing, cryostocks of ISO3-F5 were revived, subcultured, and grown anaerobically in 50-mL RM02 medium (4) supplemented with sodium formate (60 mM), sodium acetate (20 mM), methanol (40 mM), and 0.5-mL vitamin mixture and coenzyme M (10 µM), then pumped with H_2_:C0_2_ (80:20) gas mix to 1.4 bar at 39°C (5). Genomic DNA was extracted using the Qiagen Genomic-tip 20/G kit, including RNase treatment, following the manufacturer’s instructions. DNA quantity, quality, and integrity were assessed using the High Sensitivity DNA LabChip Kit on the Bioanalyzer 2100 (Agilent Technologies). The 16S rRNA gene was amplified using AmpliTaq Gold 360 master mix (Applied Biosystems) with methanogen-targeted primers 915af and 1386r (7) and sequenced on an ABI 3730 DNA system (Applied Biosystems). Strain identity was confirmed (GenBank accession number KF697734) via alignment to the National Center for Biotechnology Information (NCBI) 16S rRNA database using BLAST v2.9.0 (8). For PacBio, 30 μg of genomic DNA was fragmented using g-TUBEs (Covaris) and size-selected for fragment size of >20 kb using the BluePippin system (Sage Science). DNA fragments were end-repaired to construct SMRTbell DNA template libraries with Bioanalyzer used to assess quality and fragment size estimation. The SMRTbell DNA CLR template prep kit v2.0 (PacBio), was used for library preparation according to the manufacturer’s protocol and was sequenced using two single-molecule real-time cells on the Sequel II system v8.0, with continuous long read mode.

Read quality was evaluated using FastQC v0.12.0, and trimming of raw sequences was performed using Trimmomatic v0.39 (9). Error correction of the reads was performed as part of the Flye v2.9.1 (10), *de novo* assembly process. Quality and completeness were evaluated using CheckM v1.1.3 (11), and BUSCO v5.2.2, using the archaea_odb10 data set profile (12), respectively. The ISO3-F5 genome was annotated using NCBI’s Prokaryotic Genome Annotation Pipeline v6.6 (13), and then with the Integrated Microbial Genomes annotation pipeline v5.1.13 (14). Taxonomic assignment was based on 16S rRNA gene sequences, average nucleotide identity (15), *in silico* digital DNA-DNA hybridization analysis via the Type (Strain) Genome Server v387, and using the Genome Taxonomy Database Toolkit v2.3.0 (16). Default parameters were used for all software unless otherwise specified. The sequencing and genome features are summarized in Table 1. Its closest currently known relative is *Methanosphaera* sp. BMS from a Brahman steer (2). Close relatives of ISO3-F5 are globally distributed (1), and the ISO3-F5 genome is a valuable resource representing a potentially novel species of the genus *Methanosphaera* from ruminants.

**TABLE 1:** *Methanosphaera* sp. ISO3-F5 genomic features.

Metadata	
Strain identifier	ISO3-F5
Isolation source	Ovine rumen
Isolation country	New Zealand

Genome project information	
GenBank accession numbers	CP118753 and CP118754
BioSample ID	SAMN33419672
Assembly ID (GCA)	GCA_034480035
SRA accession number|run ID	SRX23129610|SRR27457941
GOLD Study|project|analysis IDs	Gs0161707|Gp734154|Ga0599468

PacBio data	
Total read length (bp)	1,217,386,177
No. of subreads	497,454
Max subread length (bp)	385,476
Average subread length (bp)	2,447
*N*_50_ read length (bp)	3,196
Top 10% subread length (bp)	21,153

Genome assembly statistics	
Assembly size (bp)	2,680,162
Genome coverage (×)	327
G + C content (%)	30.5
DNA replicons	2
Contig *N*_50_ (bp)	2,506,044
BUSCO C|P|M (%)^*a*^	97.9|0.5 |1.6
CheckM completeness|contamination (%)	99.2|1.6

Genome annotations (Integrated Microbial Genomes)	
No. of genes	2,421
No. of PCGs	2,360
No. of rRNAs	9
No. of tRNAs	40
PCGs with function prediction	1,742
PCGs connected to MetaCyc|KEGG pathways	557|653
PCGs with enzymes|KO|COG|Pfam	618|1,101|1,741|1,646
CRISPR array	1

Taxonomy (best match genome)^*b*^	
Assigned taxonomy	*Methanosphaera* sp.
NCBI Taxon ID	1452353
GTDB top hit (RefSeq)	M. sp003268005
ANI|AF|dDDH *d*_4_ (%)	77.7|30.4|21.9

AF, alignment fraction; ANI, average nucleotide identity; CRISPR, clustered regularly interspaced short palindromic repeats; dDDH, digital DNA-DNA hybridization; PCG, protein coding sequence.

^*a*^ C, P, and M refer to complete, fragmented or partially represented, and missing BUSCO archaeal genes (*n* = 194), respectively.

^*b*^ % identity between the ISO3-F5 and best match strain genome of *Methanosphaera* sp. BMS with GenBank accession number for genome assembly (GCA_003268005) from the GTDB, NCBI Taxonomy Database, and DSMZ collection.

## Data Availability

The complete genome sequences and associated data for *Methanosphaera* sp. ISO3-F5 have been deposited under the GenBank BioProject accession number PRJNA937785. Detailed information regarding GenBank, BioSample, Assembly and Sequence Read Archive accession numbers and Genomes Online Database (GOLD) identifiers can be found in Table 1.
